# Why We Progress

**Published:** 1896-05

**Authors:** 


### WHY WE PROGRESS.

Professors Atwater, Ross, and Wood, of Middletown, Conn., are making an experimental investigation of the nutritive value of food, under the direction of our national government. A student is confined in an air-tight room ten feet square, into which fresh air is pumped; the food is weighed before it is given, and his temperature and physical condition noted at frequent intervals. Those foods making bone, muscle, and blood best are under study.

A medical director, a place in the President's cabinet, and a national board of health are all to be realized in the near future, and why not?

When all our States have veterinary examining boards, working under similar laws and with equally high standards, then will have arrived the time for a national board, whose chief line of work should be to maintain among the several States a common minimum standard.

The increased number of cats in Pittsburg incidental to the several cat-shows over the country has become so "noticeable" as to attract the attention of the public press.

Richard Ringer, of Friend, Neb., on learning of the diagnosis of hydrophobia following the bite of a dog, seized a razor and killed himself. This shows how strong a part the mind plays in this disease, and its probable development.

One of the rules of the coming horse-show at Philadelphia reads as follows: Three veterinary surgeons will be engaged by the Association to examine all horses shown, and no prizes shall be awarded to an unsound horse in any of the saddle or harness classes, or to any horse in the breeding classes in which there exists an hereditary unsoundness; but no horse shall be disqualified under this rule unless two of the veterinary surgeons join in a written opinion, addressed to the executive committee, disqualifying it.

Dr. Samuel G. Dixon, of the Black Rock farm, near Philadelphia, is breeding several Percheron mares to hackney stallions, a cross he contends will produce a fine horse for heavy harness purposes.

Aseptolin, the lymph-product of Dr. Edson, will be given a trial covering a period of six weeks in bovine tuberculosis at Pittsburg, under the direction of veterinarian Robert Jennings, Jr. Six valuable cows will be made subjects for its trial.

The incorporated horse-shows of London will take an advance step in their efforts to better sustain the implied objects of all such organizations by having all the animals looked over in their stalls and quarters, and those not considered in the lead for prizes stood aside; those selected as close competitors for the prizes will then be subjected to a rigid veterinary examination and finally passed upon by the judges as to their merits for the various prizes offered. This should be done in every show, and not until then will these organizations fill their proper and full sphere of usefulness.

